# Shaping of Electron Beams Using Sculpted Thin Films

**DOI:** 10.1021/acsphotonics.1c00951

**Published:** 2021-11-17

**Authors:** Dolev Roitman, Roy Shiloh, Peng-Han Lu, Rafal E. Dunin-Borkowski, Ady Arie

**Affiliations:** † School of Electrical Engineering, Fleischman Faculty of Engineering, Tel Aviv University, Tel Aviv 69978, Israel; ‡ Physics Department, Friedrich-Alexander-Universität Erlangen-Nürnberg, Erlangen 91058, Germany; § Ernst Ruska-Centre for Microscopy and Spectroscopy with Electrons and Peter Grünberg Institute, Forschungszentrum Jülich, Jülich 52428, Germany; ∥ RWTH Aachen University, Aachen 52062, Germany

**Keywords:** Electron microscope, Phase masks, Beam shaping, Aberration correction, Orbital angular momentum

## Abstract

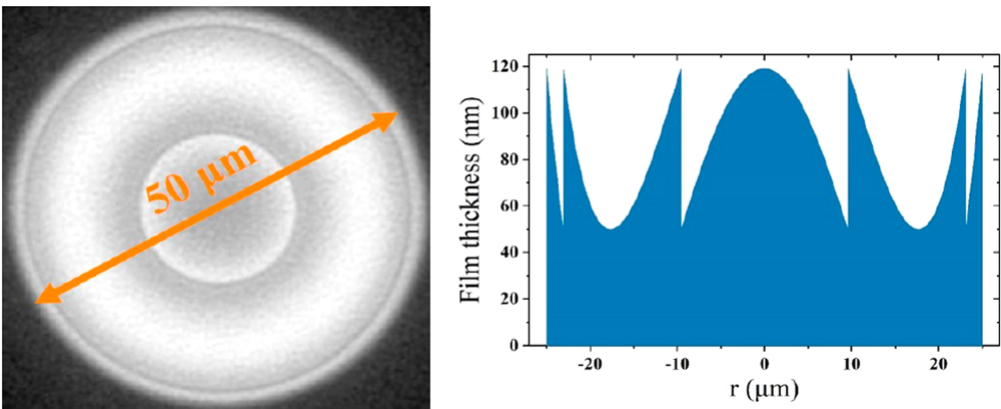

Electron beam shaping by sculpted thin films relies on electron–matter interactions and the wave nature of electrons. It can be used to study physical phenomena of special electron beams and to develop technological applications in electron microscopy that offer new and improved measurement techniques and increased resolution in different imaging modes. In this Perspective, we review recent applications of sculpted thin films for electron orbital angular momentum sorting, improvements in phase contrast transmission electron microscopy, and aberration correction. For the latter, we also present new results of our work toward correction of the spherical aberration of Lorentz scanning transmission electron microscopes and suggest a method to correct chromatic aberration using thin films. This review provides practical insight for researchers in the field and motivates future progress in electron microscopy.

Ever since the discovery of the electron, electric and magnetic fields have been used to control and manipulate electrons in free space, both spatially and temporally. In particular, electromagnetic lenses are used in all types of electron microscopes. Although rotationally symmetric lenses are the most common ones, non-rotational systems such as multipole aberration correctors can be used to correct for spherical and chromatic aberration.^1,2^ Over the past decade, the manipulation of electron beams has evolved from aberration correction to elaborate beam shaping. For example, thin film masks have been successfully used to generate electron vortex beams, which carry orbital angular momentum (OAM).^3−6^ Other approaches have involved the use of magnetic needles,^7,8^ electrostatic line charges,^9^ and magnetic lens aberrations.^10^ A recent example based on electrostatic fields is that of a programmable phase plate for electrons, which is made from an array of electrostatic elements,^11^ in analogy to spatial light modulators in light optics. Experimental and theoretical methods based on electron–light interactions have also sparked great interest. One such experimental demonstration involves the use of a high-intensity continuous laser to manipulate the electron phase.^12^ Other works suggest theoretical methods to shape electron wavepackets using interaction between electrons and short laser pulses.^13,14^ Another recent theoretical work has suggested that the aberrations of the electron lens can be corrected by creating optical near-field distributions that imprint a lateral phase on the electron wave function.^15^ A quantum mechanical treatment of the wave nature of electrons and analogies to light optics can be used to understand the above examples, as well as the following review of thin film shaping methods.

The structure of this Perspective is as follows. First, we provide the physical basis of electron optics arising from the wave nature of electrons, explain the principles of shaping electron beams using thin films, describe different mask types and compare to other shaping methods, and discuss electron beam coherence. Next, we review examples of the generation of special electron beam modes using thin film masks. In the Applications section, we show how such masks have been used for different applications such as sorting of beams according to their orbital angular momentum and structured illumination microscopy. Finally, we present new results from our study of the correction of the spherical aberration of electron lenses and suggest a possible future application of sculpted thin films for chromatic aberration correction of transmission electron microscopes. The last section summarizes this article.

## Electron Optics

We follow Voloch-Bloch et al.^5^ in the derivation of the wave function of a relativistic free electron and modify it for electron waves in matter following Reimer and Kohl.^16^ Ignoring spin effects, a relativistic free electron can be described by the Klein–Gordon equation
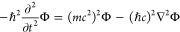
1where *m* is the electron mass, *c* is the speed of light in a vacuum, and *ℏ* is the reduced Planck constant. The electron plane wave solution of the wave function is
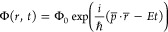
2where *E* = *γmc*^2^ is the electron energy and *p* = *γmv* is the electron momentum and they are related via the dispersion relation . In these, we use the relativistic parameters β = *v*/*c* and . In the paraxial approximation, the transverse momentum satisfies the condition *p*_⊥_ ≪ *p*, and the wave function solution becomes
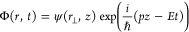
3where *r*_⊥_ represents transverse coordinates. Assuming the slowly varying envelope approximation for this solution, eq 1 is reduced to
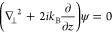
4which has the same form as the paraxial Helmholtz equation, where  is a transverse Laplacian and  is the de Broglie wavenumber of the electron. The paraxial Helmholtz equation is used to describe the propagation of light beams in the Fresnel approximation. The propagation of light beams in free space and that of an electron wave function therefore follow similar dynamics. As a result, under relevant conditions, the applications and theories of light optics can be adapted to electron optics.

It is convenient to discuss electron optics in terms of light optics and to introduce the electron “refractive index”, which is defined as the ratio between velocities in a vacuum and matter or between corresponding wavelengths, . In the absence of magnetic fields, by using the de Broglie wavelength of electrons and replacing the electron kinetic energy by *E* – *V*(*r*), where *V*(*r*) is the attractive Coulomb potential in the atom that influences the electron velocity in matter
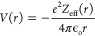
5and *Z*_eff_(*r*) is the effective atomic number that determines the dependence on *r* of the screening of the nuclear charge by the atomic electrons, the expression for the refractive index takes the form
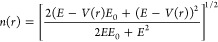
6where *E*_0_ is the electron rest energy. Assuming |*V*(*r*)| ≪ *E*, *E*_0_, this formula can be simplified to its first order approximation
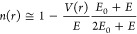
7highlighting the fact that *n* ≥ 1, as *V*(*r*) is not positive. To zeroth order, *V*(*r*) can be replaced by the constant term in its Fourier expansion, the mean value *V*_*i*_ = −*eU*_*i*_, namely, the mean inner potential (MIP). The most accurate way to measure the MIP is electron interferometry or holography.^16,17^ For electron propagation in matter, we substitute the free space de Broglie wavenumber, *k*_B_, by *k*_*m*_ = *n*(*r*)*k*_B_ in the electron wave eq 2 to obtain the relativistically corrected form of the Schrödinger equation
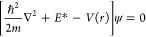
8where . For non-relativistic energies *E* ≪ *E*_0_, this equation transforms to the conventional Schrödinger equation.

An electron wave traveling in matter is often assumed to approximately experience only elastic scattering events from the atomic potentials and to accumulate a phase that is proportional to the MIP and the sample thickness. The MIP is linked to the material’s charge density distribution (see eq 5) and can therefore be used to shape the electron beam wavefront without using actual charge distributions, in analogy to light waves passing through a refractive medium and acquiring a phase shift that depends on the material’s refractive index. The optical path difference after passing through a layer of thickness *t* is Δ*s* = (*n* – 1)*t*. Therefore, the accumulated phase is
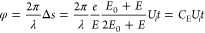
9

This physical mechanism is the basis for using thin films as electron phase plates. The values of *C*_E_ for typical transmission electron microscope (TEM) energies of 200 and 300 keV are 7.3 and , respectively. Table 1 shows values of MIP for a selection of materials that are commonly used for nanofabrication, corresponding values (in this work, for 200 keV electrons) of *t*_2π_, which is the thickness needed to obtain a 2π phase shift, and the loss from inelastic scattering. The latter is calculated using the relation
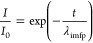
10where *I*_0_ and *I* are the intensities before and after the electrons pass a material of thickness *t*, respectively, and *λ*_imfp_ is the electron inelastic mean free path.^18^

**Table 1:** Mean Inner Potential *U*_*i*_, Thickness *t*_2π_ Required to Accumulate a 2π Phase Shift of 200 keV Electrons, and Inelastic Scattering Loss for Materials That Are Commonly Used for Nanofabrication (MIP Values Taken from refs (94 and 98))

element	*U*_*i*_ (V)	*t*_2*pi*_ (nm)	loss (%)
Si	12	71	39
Si_3_N_4_	11.3	76	43
C (amorphous)	12	71	36
Cr	22.2	39	31

## Electron Beam Shaping Using Thin Film Masks

The use of sculpted thin films to shape electron beams is becoming increasingly popular because the short electron wavelength, on the picometer scale, enables microscopy, scientific research, and material fabrication on much smaller scales than using light-based instruments. The roots of this idea were planted more than 70 years ago when, in 1947, Boersch^19^ suggested to induce a phase shift to an electron beam by using the MIP and the thickness of a material, as explained above. Experimentally, this idea was demonstrated in 1975–1976 by Willasch and Muller^20,21^ who created phase plates by building up contamination on a thin membrane. Between 1993 and 1998, Ito^22,23^ fabricated diffraction gratings and Fresnel lenses for electron beams by drilling nm-sized holes in thin films to control the electron phase. Two decades later, advances in nanofabrication and the growing use of electron beam lithography and focused ion beam (FIB) milling for shaping structures at the nanoscale have contributed to progress in this field. In 2010, Verbeeck and McMorran^3,4^ demonstrated the use of a binary amplitude mask to create electron vortex beams.

Thin films can be utilized to shape both the amplitude and the phase of an electron beam. The spatial amplitude modulation, *A*(*x*, *y*), and the phase accumulation, φ(*x*, *y*), that an electron plane wave acquires when passing through a thin film mask define its transmittance
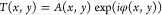
11

Amplitude masks spatially modulate the electron wave amplitude while inducing a spatially constant phase. Practically, they are often made of highly scattering materials, in which areas are opened to allow local transmission through the film, in order to create binary amplitude profiles. In phase masks, varying thickness profiles or combinations of different materials are used to produce spatially varying phase shifts (eq 9), while maintaining a constant amplitude modulation. Mixed types of masks can also be obtained if both the amplitude and the phase in the transmittance term vary spatially.^24^

## On-Axis and Off-Axis Masks

Harvey et al.^24^ explain in detail how the complex electron beam amplitude is diffracted when propagating through a mask that has a periodic design. The signal efficiency, which is defined as the ratio between the *m*th diffraction order and the electron beam hitting the mask, , may vary between different mask types and designs and should therefore be chosen carefully. Masks that are designed for the zeroth order are called “on-axis” masks, while “off-axis” masks are used to obtain a desired beam at *m* ≠ 0 and therefore contain a carrier wave term in their complex amplitude, , which is dependent on the local periodicity.

## Comparing Thin Film Masks to Electromagnetic-Based Shaping Methods

One of the greatest advantages of thin film masks is the ability to precisely modulate the electron phase anywhere in the plane of the film by varying its thickness. Such nearly arbitrary modulations, which may include sharp jumps, cannot be realized with magnetic multipoles or electrostatic means because the static magnetic and electrical fields in free space must be continuous. Furthermore, thin film masks are technically simple to use. While the above-mentioned concepts of electron beam shaping by electromagnetic manipulation of electrons require external instruments and modifications to the microscope, such as lasers, current induction, spatial light modulators, etc., thin film masks are passive devices that require only simple installation at one of the existing microscope apertures without any other modifications. Moreover, the common method of milling thin films to create masks with thickness variability at the nanometer scale allows great tunability, mainly for phase manipulation that is directly dependent on the thickness of the film. It is practical to insert different milled masks in the microscope all at once to conduct various measurements. One drawback, however, is the loss of signal contrast due to elastic and inelastic scattering from the membranes themselves, sometimes so large that measurements are significantly affected. These losses are not present in electromagnetic multipole correctors, since the electron beam passes in a vacuum.

## Electron Beam Coherence

Throughout this article, we consider a temporally and spatially coherent beam. Temporal decoherence originates from the source energy spread, and spatial decoherence is caused by the source size. It is convenient to explain their significance by considering the electron beam’s probe size at the focus. It is affected by diffraction, chromatic and spherical aberrations, and the demagnified source size. Here, we use the 50% current-enclosed criterion to calculate the diameter of each contribution and combine them using the root power sum method.^25^

The temporal decoherence is manifested by the contribution of the chromatic aberration to the spot size. It is given by , which depends on the chromatic aberration coefficient, *C*_c_, the convergence semiangle, α, and the energy spread, Δ*E*/*E*, arising from the source emission spectrum. *C*_c_ of the probe forming lens in a typical TEM is in the order of 1–2 mm, and together with the small energy spread of modern field emission gun sources, Δ*E* < 0.7 eV, this contribution is rather insignificant. Hence, temporal decoherence in most systems is very small. However, energy filtering using a beam monochromator is a common method to decrease this decoherence even more when desired. In the Applications section of this Perspective, we propose the concept of chromatic aberration correction using thin films which may improve the spatial coherence by reducing *C*_c_, which may be very large in certain imaging modes.

Spatial coherence is demonstrated by the contribution of the demagnified source size, , where *I* is the beam current at the specimen and *B* is the beam brightness which depends on the source size. In modern field emission gun sources, the high brightness values, in the order of 10^12^–, make this contribution negligible and spatial decoherence insignificant. We note, however, that the effects of the spatial decoherence may become significant with other electron sources, such as thermionic emitters.

## Generation of Special Electron Beam Modes

During propagation in a vacuum in a microscope column, an electron beam obeys the time-independent Schrödinger equation, which is identical in form to the paraxial Helmholtz equation, as explained in the electron optics section (eq 8 with *V*(*r*) = 0). In light optics, self-similar solutions of this equation, such as Hermite–Gauss and Laguerre–Gauss modes,^26^ are routinely used in applications to optical communication.^27^ Other examples include Airy^28,29^ and similar accelerating beams,^30^ as well as non-diffracting solutions such as Bessel beams.^31,32^ Superoscillating beams,^33^ which contain lobes with dimensions that are much smaller than a diffraction-limited spot, have also been realized with electron beams.^34^ As a result of the proliferation of phase masks, including electrostatic and magnetic devices for beam shaping in electron optics,^35^ such special beams are destined to become applicative tools for the investigation of the fundamental properties of matter.

Electron vortex beams are considered in both theoretical and experimental investigations^6^ as prominent candidates for interactions with matter in the TEM. Such beams approximate the precise Laguerre–Gauss solution of the wave equation while maintaining its most interesting property, orbital angular momentum.^36^ By virtue of the chirality of electron vortex beams, shaped atomic-sized probes were expected to be able to measure electron energy-loss magnetic dichroism. However, this elusive property is yet to be measured satisfactorily.^37^ Furthermore, it is well-known that the spin magnetic moment of an electron *μ*_s_ is related to the electron spin angular momentum through the Bohr magneton, *μ*_s_ = −*gμ*_B_*S*_*z*_/*ℏ*, where *g* is the gyromagnetic ratio and *S*_*z*_ is the projection of the angular momentum on *ẑ*. In contrast, the dipole magnetic moment of a vortex beam depends on its OAM charge *l*, such that its projection on *ẑ* is *μ*_L_ = −*μ*_B_*L*_*z*_/*ℏ*. Therefore, the total magnetic moment of a vortex beam can be very large.^38,39^

Electron vortex beams have been shown to provide the means to map the out-of-plane magnetic field, parallel to the beam propagation direction, in matter,^40,41^ which cannot be achieved using non-structured beams. Numerical investigations of electron vortices propagating through amorphous materials have been performed.^42^ Recently, methods to generate OAM using amplitude masks and laser-assisted multiphoton ionization^43^ have been compared. The shaping of an electron probe into an electron vortex beam in a scanning electron microscope (SEM), rather than a TEM, has also been shown.^44^

Interestingly, Laguerre–Gauss solutions, which are circular-symmetric, can be converted into Cartesian-coordinate-based Hermite–Gauss solutions, and vice versa, by applying astigmatic phase terms.^45,46^ This conversion has been demonstrated in experiments in the TEM^47−49^ and could be used as a method to generate or measure high-purity vortex beams, as explained in the electron vortex sorter section. Experimentally, Hermite–Gauss approximations can be generated by phase masks^50^ but have also been generated by a magnetic needle and used to investigate the potentials of localized surface plasmons.^51^

Airy beams have already been demonstrated experimentally in the TEM.^5^ Although their accelerating property can be explained geometrically by a clever superposition of rays forming a caustic, it is fascinating to realize that the observation of an Airy beam in a TEM is a practical approximation of a unique solution of the Schrödinger equation: a non-diffracting, accelerating free-electron wavepacket.^28^ Another non-diffracting beam, namely, the Bessel beam, has also been generated by various means.^52−54^ This beam is expected to be resistant to spherical aberration in the TEM^55^ and has been employed to measure nanoscale strain in a Si/SiGe layer stack.^56^

**Figure 1:**
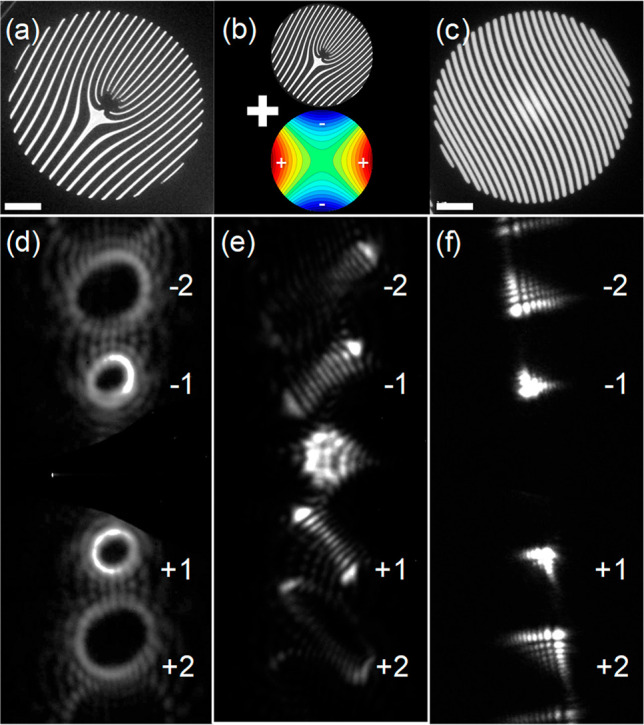
Selected examples of electron beam shaping masks and measurements. (a) Fork mask generating vortex beams with an OAM of 10ℏ. (b) As for part a, using the microscope stigmator to apply a transformation to a Hermite–Gauss-like beam. The colored circle depicts the magnetic field distribution of a quadrupole lens, acting as a stigmator. (c) Off-axis Airy mask. (d) Measurement of part a in a diffraction plane, showing vortex beams around the central, transmitted beam (mechanically blocked to protect the camera). (e) As for part d, after engaging the TEM’s built-in stigmators. In a diffraction plane, the Laguerre–Gauss vortex approximations have transformed into a Hermite–Gauss-like intensity pattern of order 10, as evidenced by counting the dark lines. (f) Airy beams measured in the diffraction plane, resulting from the mask in part c. The scale bars in part a–c are 2 μm. The diameter of the masks is 10 μm.

In Figure 1, we show examples of masks for generating vortex (a, d), Hermite–Gauss-like (e), and Airy (c, f) beams and associated measurements. Parts a and d of Figure 1 show a vortex fork mask of order 10 and a corresponding diffraction pattern, respectively. Figure 1b depicts the usage of TEM stigmators, which approximate a quadrupole magnetic field pattern through which the electrons propagate. This field acts similarly to a cylindrical lens in light optics, transforming a vortex Laguerre–Gauss-like beam into a Hermite–Gauss-like beam,^48^ whose intensity pattern is measured in a diffraction plane in Figure 1e. In Figure 1c, we present an off-axis Airy mask. In this case, the cubic phase imparted to the incoming beam is translated to off-axis separated orders of Airy beams, as shown in Figure 1f.

## Applications

We now review recent works and results demonstrating practical technological uses of electron beam shaping by thin film phase masks.

### Electron Vortex Sorter

With increasing research into electron beams that carry OAM, the development of techniques for measuring and analyzing OAM modes has naturally ensued. The initial approach, studied by Guzzinati et al.,^57^ involved the application of binary masks, such as fork gratings placed in the projection system, and measurement of the far-field diffraction pattern using a CCD camera. If discrimination between vortex and non-vortex beams was desired, then a pinhole was placed at the diffracted beam position. The problem with this method was that the fork grating mask and pinhole reduced the signal intensity greatly, already in the first diffraction order and more so for the higher orders. Moreover, the higher the OAM selectivity (i.e., the smaller the pinhole), the lower the signal. Two other binary mask methods employed by Guzzinati et al. involved the use of a triangular aperture and a knife edge, each of which had its own advantages and limitations. However, these methods were only applicable to eigenstates of OAM, or some cases of incoherent superpositions of these states, but did not translate well to arbitrary beams, for which discrimination between OAM and the form of the beam itself is required.

A recent development in this field is that of electron beam sorters, which are used to measure the spectrum of OAM modes in a beam, based on the sorting principle of OAM in light optics.^58^ In this scheme, polar coordinates are transformed to Cartesian coordinates, so that azimuthal phase changes originating from different OAM modes are converted into lines, with the different modes separated spatially. Experimental success in OAM sorting was achieved, for example, by using electrodes.^59^

**Figure 2:**
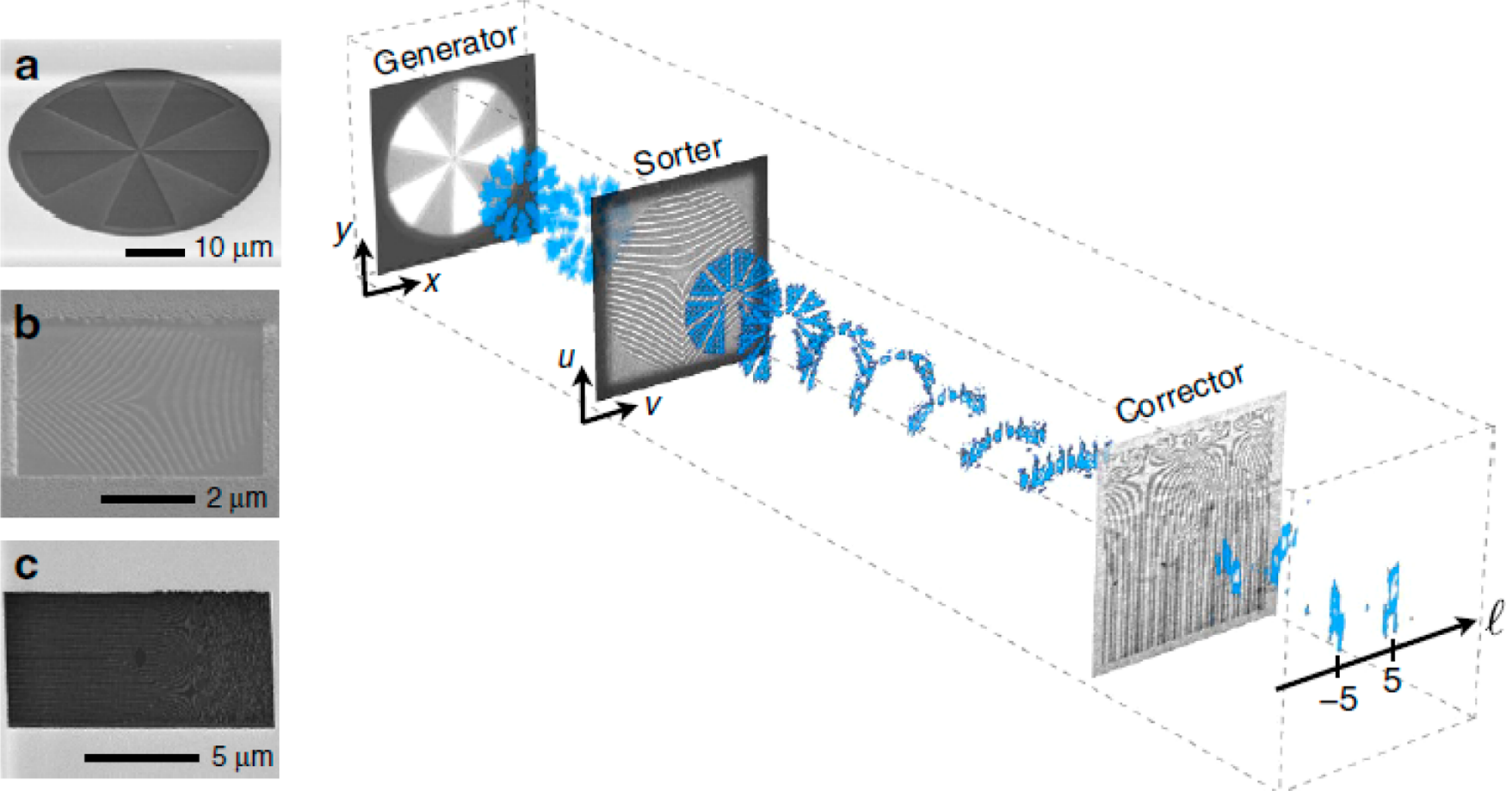
Principle of operation of an OAM sorter. (a–c) SEM images of the generator, transformation, and corrector holograms. Taken from ref (60).

Here, we discuss a thin film approach demonstrated by Grillo et al.^60^ Similar to an optical approach that involves the use of two lenses to perform the transformation, they used two thin film phase holograms, one to map the azimuthal phase change onto transverse Cartesian lines and a second to correct for phase distortions introduced by the first mapping hologram. They demonstrated the process for various superpositions of up to 10 OAM states and analyzed the magnetic properties of a magnetized sample. Figure 2 illustrates the sorting process and shows SEM images of the thin film holograms. The initial TEM beam first passes through a generator hologram placed in the condenser lens aperture and modifies it to carry OAM. The beam then propagates through the sorter, with the first phase hologram, which performs the coordinate transformation ((*x*, *y*) → (*u*, *v*)), placed in the TEM sample holder. The corrector hologram is positioned in the selected area diffraction (SAD) aperture plane. Finally, the resolved OAM spectrum is captured on a CCD camera. Grillo et al. concluded that this method yielded more information about the beam’s phase than existing sorters. They suggested to improve the effectiveness of their sorter by substituting the holograms with electrostatic fields to reduce absorption. In 2021, Tavabi et al.^61^ demonstrated such an electrostatic sorter.

### Volta Phase Plates

Phase contrast in TEM is a key tool for high-resolution measurements. The TEM contrast transfer function (CTF), sin(χ(*k*)), describes the effect on the phase shift of aberrations that include defocus and spherical aberration. The wave aberration function takes the form
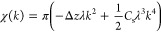
12where Δ*z* is the defocus and *k* is the spatial wavenumber, which describes the deflection angle of the electron beam from the optical axis of the microscope. At high *k* values, the CTF is oscillatory and changes sign rapidly, while phase contrast is also suppressed at low spatial frequencies, limiting the obtained information. Working at certain defocus values may flatten the CTF over some interval, but it does not allow it to be expanded beyond the information limit. A popular solution, realized by Danev et al.,^62^ is to apply a Zernike type^63^ phase plate (ZPP) in the back focal plane of the TEM objective lens. The back focal plane, under ideal conditions, can be treated as the Fourier (spatial spectra) plane, in which different frequencies or *k* values can be resolved. Phase plates are commonly made from thin amorphous carbon films, which are designed to introduce a constant π/2 phase shift to the scattered (*k* ≠ 0) part of the beam, while allowing transmission of the unscattered (*k* = 0) beam through a small hole in the center. The smaller the hole, the higher the contrast. However, diffraction from the hole edges results in a fringe pattern around image features, which is difficult to perfectly demodulate.^64,65^ Danev et al.^66^ described an alternative electron-beam-created phase plate, which is referred to as a Volta phase plate (VPP) and modifies the CTF without diffraction fringes. They used a heated (>100 °C) carbon film without a hole, on which the interaction area with the non-diffracted electron beam resulted in a phase change to the electron beam itself. The interaction is assumed to cause local changes to the inner or surface potentials of the carbon film. The resulting Volta potential causes phase shifts in the electron beam, which can be used for enhancing image contrast. The effect was discovered while testing materials for use as ZPPs and is thought to be responsible for the aging of masks. However, when it is used as the generator of the phase shift, the corresponding fabricated masks last longer and can be reused. For the phase shift to originate only from the Volta potential, a thin film is required, in which modifications to the inner potential are small enough to be neglected.

**Figure 3:**
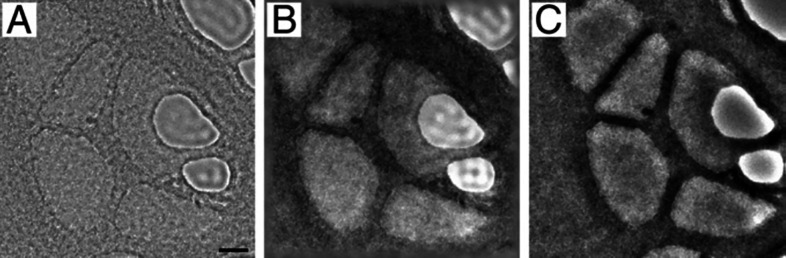
Images of a lacey carbon film recorded using (A) a ZPP and (C) a VPP. The image in part B is a fringe-reduced software-filtered version of the ZPP image in part A. Scale bar: 20 nm. Taken from ref (66).

Figure 3 shows a comparison between ZPP images recorded before (Figure 3A) and after (Figure 3B) fringe reduction, as well as a VPP image (Figure 3C) of a lacey carbon film. The VPP image has higher contrast and fewer fringes than the ZPP images.

### Structured Illumination for Phase Contrast Scanning TEM

The recent advent of fast direct electron detectors^67^ has revolutionized both cryo-electron microscopy for three-dimensional reconstruction of biological macromolecules^68^ and momentum-resolved scanning TEM (STEM)^69^ for strain mapping,^70^ electromagnetic field mapping,^71^ and phase contrast imaging.^72,73^ Momentum-resolved STEM refers to the acquisition of 2D convergent beam electron diffraction (CBED) patterns in momentum space, over a 2D matrix of scanned electron probe positions in real space. This technique is often referred to as 4D-STEM because the data are four-dimensional. It offers a unique opportunity to manipulate and analyze a flexibly selected range of scattering information for each sample position. In particular, one can retrieve the phase of the exit wave function from CBED intensity images (in which the phase appears to be lost) because of redundant information in the 4D data set. Recently developed methods also take advantage of structured illumination (or a structured probe) to fulfill the optical requirements or to extend the application range.

**Figure 4:**
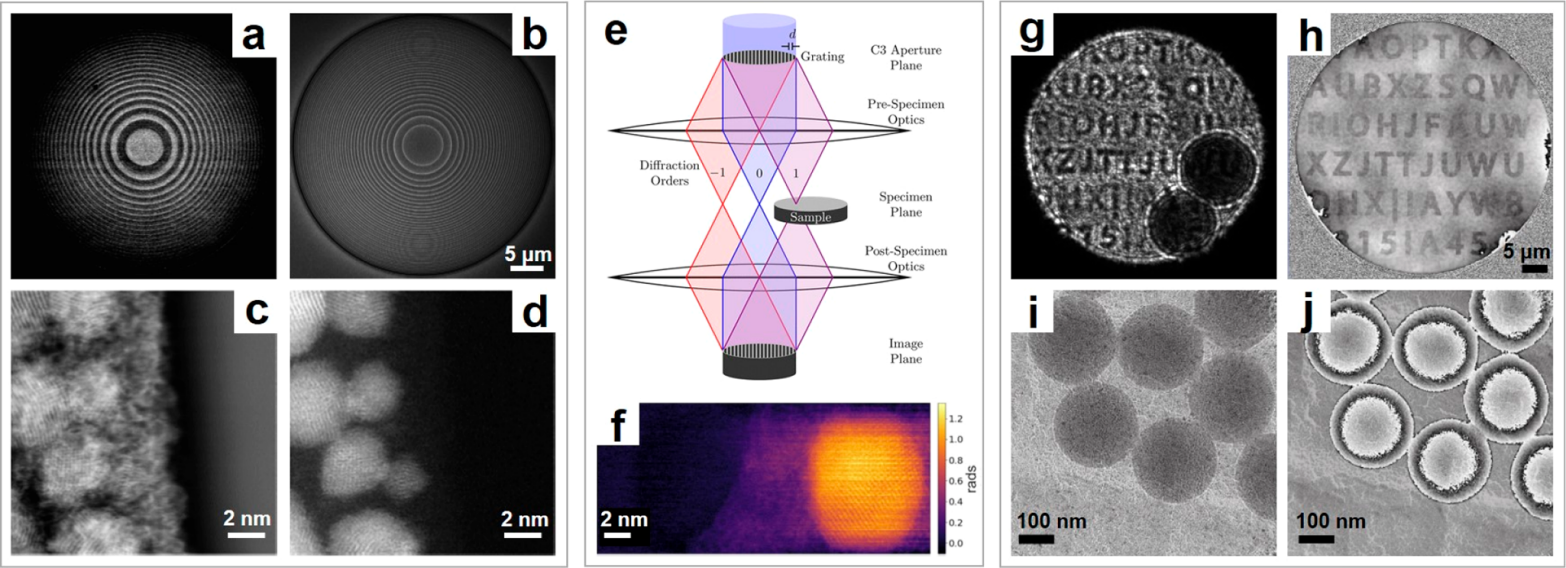
Structured illumination for phase contrast STEM. (a–d) MIDI-STEM. (a) Example CBED pattern consisting of alternating constructive and destructive interference. (b) SEM image of the patterned Fresnel zone plate. (c, d) Virtual MIDI-STEM and ADF images of Au nanoparticles on a carbon support, processed from the 4D-STEM data set. Taken from ref (75). (e, f) STEM holography. (e) Schematic diagram of the optical setup, which involves the use of a diffraction grating in a condenser aperture position. (f) Reconstructed phase image of Au nanoparticles on a thin carbon support. Taken from ref (80). (g–i) Near-field electron ptychography. (g) An example near-field diffraction pattern. (h) Reconstructed phase image of the diffuser. (i, j) Reconstructed amplitude and phase image of the latex sphere on carbon/Au nanoparticle substrate, processed from only nine diffraction patterns. Adapted from ref (90).

The first example is taken from so-called annular differential phase contrast (ADPC) imaging,^74^ or matched illumination and detector interferometry (MIDI-STEM).^75^ The concept dates to 1974^76^ and is based on the alignment of alternating diffraction rings in CBED with oscillations in the contrast transfer function of an electron probe. Although it was originally proposed to realize such a probe by adjusting the defocus and spherical aberration,^77^ this significantly limits the number of alternating rings (or zones). Recently, it has been demonstrated experimentally by sculpting a thin film into a 20-ring Fresnel zone plate (Figure 4b) with alternating 0 and π/2 phase shifts,^78^ resulting in constructive and destructive interference on the detector (Figure 4a), which are later integrated separately by applying a virtual detector to the 4D-STEM data set (matching the illumination with the detector). Subtraction of these two signals removes the nonlinear information and provides improved phase contrast. The approach is demonstrated in Figure 4c,d, which shows a MIDI-STEM image and an ADF image, from the same region of a carbon-supported Au nanoparticle sample. The MIDI-STEM image shows much stronger contrast from the carbon support than the ADF image, which is better suited to visualize heavier elements. The method has recently been extended to thicker specimens for optical sectioning.^74^

Thanks to advanced nanofabrication, this concept can be revived after 45 years. The second example involves the use of an even simpler thin film mask—a Bragg diffraction grating—to implement an electron interferometry technique that is referred to as STEM holography.^78^ Historically, the technique required the use of an electron biprism (a charged wire) in the illumination optics to split the electron beam, with one part being focused on the specimen and the other part allowed to propagate through a vacuum in the form of a reference wave. Downstream in the column, they were allowed to interfere with each other to form fringes (also known as holograms). Both the amplitude and the phase of the specimen could be reconstructed from recorded holograms. Instead of installing a biprism into the illumination system, a carefully prepared amplitude-dividing diffraction grating can be used to fulfill the same goal. It can be inserted into the condenser aperture position of almost any electron microscope (Figure 4e). In combination with a fast 4D-STEM detector, this optical setup has been demonstrated experimentally to provide interpretable phase contrast^79^ and even to achieve atomic spatial resolution in phase images (Figure 4f).^80^

One way to make full use of 4D-STEM CBED information is ptychography. This technique is based on the measurement of correlated CBED patterns across substantially overlapped sample positions, which allows for computational reconstruction of both the phase and the amplitude of an object. It was proposed more than 50 years ago^81^ but was not widely adopted in electron microscopy until the recent introduction of fast detectors, despite several historical demonstrations.^82,83^ As a result of the development of improved reconstruction algorithms, ptychography has been developed in several directions, in order to overcome the diffraction-limited resolution,^73,84^ to reduce the electron dose budget,^85,86^ and to extend the technique to 3D imaging.^84,87^ A defocused nm-sized probe is usually used to sample real space efficiently, while providing inhomogeneous sampling in reciprocal space. Alternatively, a structured electron probe with random phase vortices^88^ can be used to produce a nm-sized probe for real space sampling, while remaining in focus and providing a range of incident beam angles at the same time. Simulations show that such a structured probe introduces strong fluctuations in a CBED pattern, which boosts its performance for low-dose reconstruction.^89^

Recently, a variant of ptychography, near-field electron ptychography (NeeT), has benefited from the introduction of sculpted thin film masks.^90^ This setup does not involve the recording of far-field (i.e., Fraunhofer) CBED patterns, as in most ptychography experiments. Instead, it involves the acquisition of near-field (i.e., Fresnel) diffraction patterns, which look like defocused images (Figure 4g). The use of a random phase mask (also referred to as a diffuser) can expand the usable aperture diameter and, thus, the field of view for each scanning position, which allows for very efficient large field of view phase mapping. Instead of the hundreds of diffraction patterns that other approaches may require, only nine diffraction patterns are sufficient to reliably reconstruct the amplitude and phase of the latex spheres shown in Figure 4i,j. In this example, the latex spheres are dispersed on a carbon/Au nanoparticle substrate. A field of view of ∼1 μm^2^ can be achieved with better than 4 nm spatial resolution. With a reasonable number of diffraction patterns, the reconstructed field of view can be extended to more than 100 μm^2^ with the same spatial resolution.

An additional advantage of ptychography is that it can simultaneously recover the transmission function of the illumination, i.e., the diffusers in this case. As shown in Figure 4h, the reconstructed phase of the diffuser matches very well with the designed value apart from a bit of low-frequency phase gradient at the edges which may be due to residue aberration or electrostatic charging of the diffuser. In fact, the measured maximum phase is only 3% deviated from the design value, which proves the FIB fabrication quality but also manifests that this can be used to calibrate the phase shift of the sculpted thin film masks during operation.

### Spherical Aberration Correction

In transmission electron microscopy, a major resolution limitation originates from the positive spherical aberration induced by the circular structure of the magnetic objective lens,^91^ preventing it from focusing the electron beam to a diffraction-limited spot. Over the past 25 years, aberration corrector devices based on multipole lens systems have been in use.^1,92,93^ However, they are expensive to produce and require modifications to a TEM column for installation. An alternative approach of using thin film electron phase plates to correct for spherical aberration in STEM has recently been demonstrated.^94^ In this work, the thin film corrector enabled atomic resolution in HAADF STEM which was shown by resolving the 136 pm separation of atomic silicon dumbbells. The reported probe size measurement (full width at half-maximum) was improved from 183 pm (uncorrected) to 124 pm (corrected). Such a device is simpler to produce, requires fewer modifications to the system, and is orders of magnitude cheaper. Moreover, while multipole correctors cannot be installed in existing non-corrected microscopes, thin film correctors provide a plug-and-play tool for such systems.

Spherical aberration results in deviation from a spherical wavefront, such that the farther rays pass from the optical axis, the shorter the distance they are focused onto. As a result, they are not focused to a singular, diffraction-limited point in the focal plane but to a finite circular spot. The principle of thin film aberration correction is to introduce a phase shift that is inverse to that of the aberration itself, in order to cancel the deviation from the spherical wavefront. This approach can be realized by placing a corrector membrane in the lens aperture, where the beam is collimated. The corrector’s overall thickness depends on Λ, the dimensionless figure of merit “peak-to-valley” (PTV)^95^ which describes the required number of cycles of 2π phase shift and can be calculated from the expression
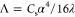
13where *C*_s_ is the spherical aberration coefficient of the lens, α is the convergence semiangle, and λ is the de Broglie wavelength of the electrons. The phase shift resulting from spherical aberration and defocus is
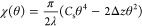
14where θ is the beam semiangle to the optical axis and Δ*z* is the defocus. If Scherzer defocus,^96^ Δ*z* = 0.5*C*_s_*α*^2^, is chosen to minimize the spherical aberration at the aperture edge, then the resulting thickness profile is
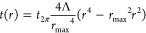
15where *r* is the radial coordinate and *r*_max_ is the radius of the corrector, which acts as the beam aperture. Large Λ values are required for the correction of large spherical aberrations but result in thick correctors that cause significant inelastic scattering (which effectively changes the energy and direction of the electrons) and poor transmission of electrons passing through them. These limitations can, in turn, lead to unwanted background noise and decreased contrast. Overcoming these effects in a “continuous” corrector may be possible by using a “fractured” design, in which the accumulated phase resets to zero every 2π. This approach limits the corrector thickness to *t*_2π_, according to the following thickness profile:
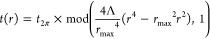
16

By extending the method presented in ref (94), we fabricated and tested a fractured corrector design for STEM mode on a 200 kV Thermo Fisher Scientific (TFS, formerly FEI) Talos F200X microscope. This microscope is not equipped with a multipole aberration corrector. The use of a “fractured” thin film corrector allows for the correction of spherical-aberration-induced blurring while maintaining reasonable contrast. The inelastic-scattering-induced intensity loss would be reduced by 20–40% depending on the constant thickness support layer of the corrector. We tried to apply a maximum phase shift of 4π in such a thin film corrector. Thanks to the “fractured” design, the thickness difference only goes up to *t*_2π_. As for many non-aberration-corrected microscopes, it only has two condenser lenses (apart from the mini-condenser lens) which prevents users from varying the convergence semiangle flexibly, as in a three-condenser-lens STEM. We developed a protocol to realize this, in order to exploit the thin film corrector at a larger convergence angle for a given aperture diameter. The technical details will be reported in depth elsewhere.

Due to the noisy background from inelastic scattering, the performance of such devices is not comparable to state-of-the-art multipole correctors. However, we have found a case where thin film correctors provide a unique technological solution for non-corrected systems as well as many existing multipole-corrected systems. We extended the concept to correct for the spherical aberration of field-free STEM mode on a 300 kV TFS Titan G2 microscope^97^ by using a continuous design. This microscope has a multipole aberration corrector but only for the post-specimen imaging optics and not for the pre-specimen illumination optics. The STEM mode, including field-free STEM, therefore remains affected by spherical (as well as chromatic) aberration. Field-free STEM (also referred to as Lorentz STEM) is a technique that is used to image magnetic materials by turning off the objective lens and focusing the beam using a condenser lens that is situated farther from the sample, in order to avoid subjecting it to magnetic fields. Currently, in many existing electron microscopes, owing to the limited strength of the mini-condenser lens (which is close to the objective lens), it is not possible to work in aberration-corrected Lorentz STEM mode, even when a conventional multipole corrector is implemented in the pre-specimen illumination optics, making it impossible to study magnetic specimens in STEM mode at high spatial resolution. Moreover, the Lorentz STEM mode suffers from much larger spherical (as well as chromatic) aberration because of the longer focal length of the condenser lens that is used to focus the probe. Therefore, its spatial resolution is limited. The typical spherical aberration coefficient in the Lorentz STEM mode can be a few tens of (or even more than 100) meters, while that in the conventional high-resolution STEM mode (with the objective lens turned on) is usually 0.5–3 mm, i.e., 4 orders of magnitude smaller.

**Figure 5:**
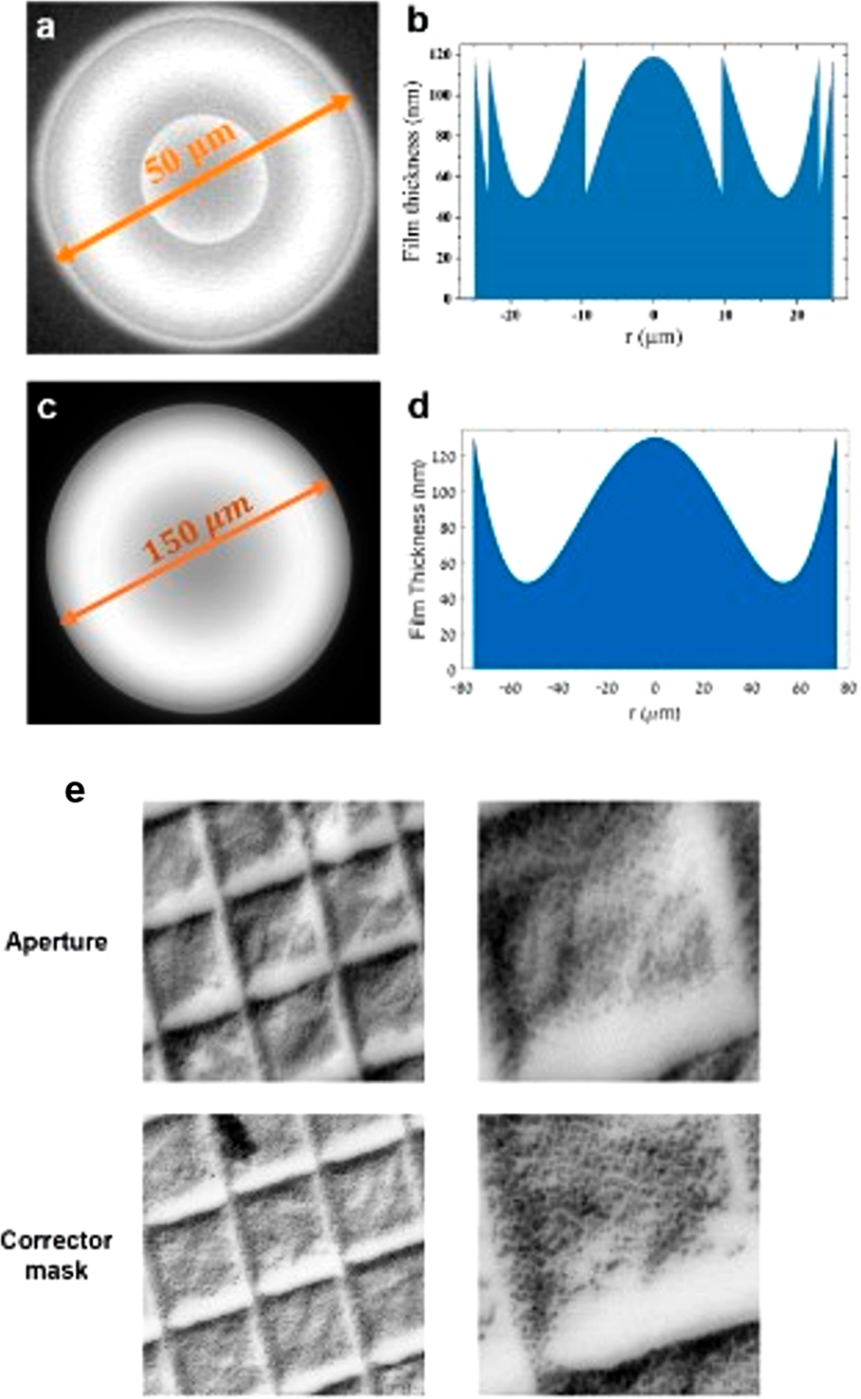
(a, c) Images of fabricated correctors and (b, d) corresponding designed radial thickness profiles. (a, b) Fractured corrector of diameter 50 μm for the conventional high-resolution STEM mode with Λ = 2 and *t*_2*pi*_ = 69 nm. (c, d) Continuous corrector of diameter 150 μm for the Lorentz STEM mode with Λ = 1.05 and *t*_2*pi*_ = 77 nm. (e) A comparison between low-magnification STEM images of the Au waffle grid using a 150 μm aperture and the corrector mask shown in part c. Both were taken at α = 1.3 mrad. One can see many more details on the Au surface with the corrector.

Here, similarly to the approach used in ref (94), thin film correctors were fabricated using FIB milling on Au-coated silicon nitride films that had thicknesses of 100–200 nm. Such silicon nitride films are mechanically robust, are commercially available, and can be readily patterned to a chosen thickness profile for phase shaping. The Au coating is only used as a diaphragm to block the electrons. Figure 5 shows recently fabricated correctors, in the form of images (a, c) and corresponding designed radial thickness profiles (b, d). Figure 5a,b shows a fractured corrector with a diameter of 50 μm designed for the conventional high-resolution STEM mode with a Λ value of 2 cycles (i.e., 4π phase shift) and *t*_2*pi*_ of 69 nm. Figure 5c,d shows a continuous corrector for the Lorentz STEM mode with Λ = 1.05, *t*_2*pi*_ = 77 nm, and a diameter of 150 μm. Figure 5e shows the resolution improvement achieved by using this membrane. We took corrected and uncorrected images of a Au waffle grid in low-magnification STEM (field free). Clearly, the image taken using the corrector mask provides sharper details with respect to the uncorrected image.

Contamination is a notable issue of thin film phase plates that are placed at the back focal plane of the objective lens. It can affect the performance and charge alleviation of the phase plates and may reduce their functionality over time. Thin film spherical aberration correctors, however, are exposed to a much smaller current density (about 10 orders of magnitude smaller) because of their placement in the Condenser 2 aperture. We found that our correctors are robust, that their performance did not degrade in over 100 h of use, and no cleaning was needed in order to maintain their functionality.^94^ Furthermore, we have not experienced charging issues using our correctors.

### Proposal for Chromatic Aberration Correction Using Thin Films

**Figure 6:**
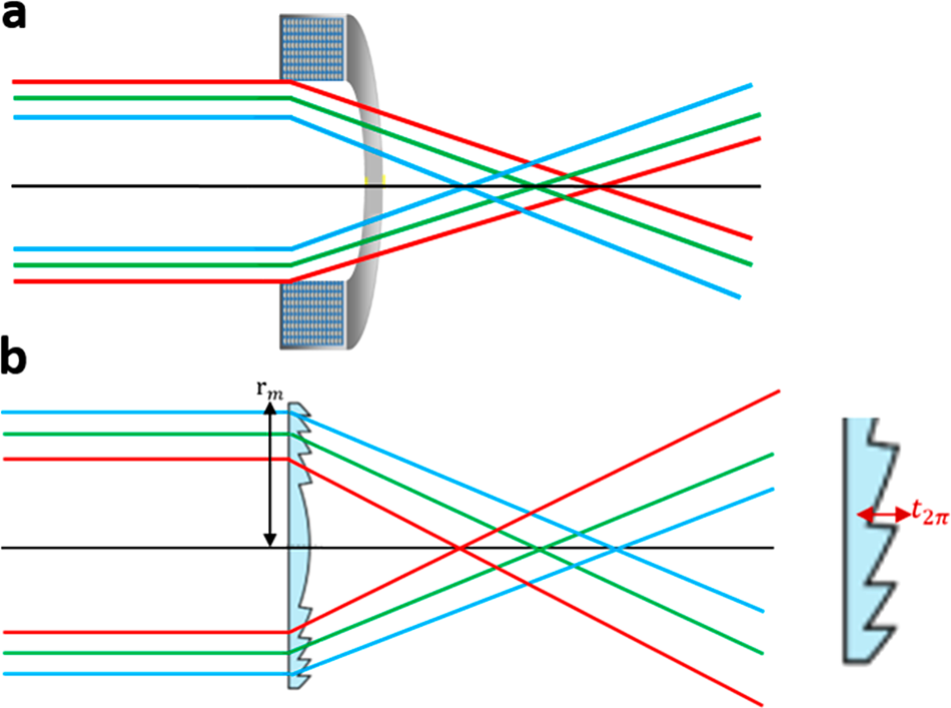
Schematic illustrations of the chromatic abserration of (a) an electron magnetic lens and (b) an electron diffractive lens fractured design. *r*_*m*_ is the radius of the *m*th concentric annular section. The peak-to-valley thickness, *t*_2__π_, introduces a 2π phase shift to the electron beam. Different rays’ colors represent the wavelength spread of the electron beam. (a) Longer wavelengths have a larger focal length. (b) The thin film lens is designed to have the opposite chromatic aberration. Combined together, the diffractive lens’ negative chromatic aberration cancels the aberration of the magnetic lens.

Chromatic aberration, which causes the focal length of a lens to be wavelength-dependent (illustrated in Figure 6a), usually has little effect on the focused probe size when compared with spherical aberration in STEM mode due to the small energy spread of modern TEM gun sources (Δ*E* < 0.7 eV). However, it may become significant in a spherical-aberration-corrected system, limiting the probe from becoming diffraction limited. In addition, in Lorentz STEM mode, the chromatic aberration coefficient can be on the order of ∼10^2^ mm, i.e., 2 orders of magnitude larger than for regular STEM imaging. In light optical systems, diffractive elements are used to compensate for chromatic aberration of refractive elements by inducing the same aberration but with the opposite sign. In analogy, we examined the possibility to correct chromatic aberration of magnetic lenses by designing an electron diffractive lens, which is based on Fresnel lens principles in light optics, as shown in Figure 6b.

We consider a fractured design with *m* periods of the electron wavelength, λ, where *r*_*m*_ is the radius of the *m*th concentric annular section in the lens. From this, together with the approximated wavefront sag , we conclude that
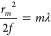
17where *f* is the focal length of the diffractive element. We can write
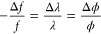
18in terms of the lens power. From this and the requirement that the difference in the power of the magnetic lens, Δ*ϕ*_*M*_, which results from chromatic aberration, will be compensated completely, we evaluate *r*_*m*_ to be
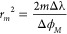
19where Δλ is determined from Δ*E*, the energy spread of the electron gun, according to , where . The maximal thickness of the elements, *t*_2π_, introduces a 2π phase shift to the electron beam. Considering the focal length of the focusing lens, *f*_*M*_, and its deviation due to chromatic aberration, Δ*f*_*M*_, where Δ*f*_*M*_ ≪ *f*_*M*_, Δ*ϕ*_*M*_ can be expressed as . Δ*f*_*M*_ was evaluated to be , where (94) is the contribution of chromatic aberration to the probe diameter. The radius of the *m*th section is then
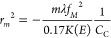
20

With similarity to thin film spherical aberration correctors, a chromatic aberration corrector for the objective lens would be installed in the Condenser 2 lens aperture as an addition to the STEM column. A design for typical Lorentz mode STEM parameters, such as *C*_c_ = 200 mm, *E* = 200 keV, *ΔE* = 0.7 eV, and a lens aperture diameter of 50 μm, consists of *m* ≈ 1600 sections of uneven width with an average feature size of ∼15 nm and is difficult to fabricate.

## Outlook and Summary

In this Perspective, we have reviewed recent work on shaping electron beams using sculptured thin films based on amplitude and phase manipulation of the electron wavefront. Such masks were used to generate different kinds of OAM-carrying electron beams and to measure or sort their momentum states, as well as for high-resolution TEM applications such as spherical aberration correction in conventional high-resolution and Lorentz STEM modes and for phase contrast electron microscopy including phase plates for contrast enhancement and structured illumination for a variety of phase contrast STEM techniques. Potential applications of electron thin film masks require nanofabrication milling abilities on the nm scale, which may be feasible with future technological advances. A highly valuable achievement may be the correction of chromatic aberration in STEM using thin films, for which we have suggested a possible mask design.
